# Technological Parameters, Anti-*Listeria* Activity, Biogenic Amines Formation and Degradation Ability of *L. plantarum* Strains Isolated from Sheep-Fermented Sausage

**DOI:** 10.3390/microorganisms9091895

**Published:** 2021-09-07

**Authors:** Nicoletta P. Mangia, Michele Cottu, Maria E. Mura, Marco A. Murgia, Giuseppe Blaiotta

**Affiliations:** 1 Dipartimento di Agraria, University of Sassari, 07100 Sassari, Italy; mcottu@uniss.it (M.C.); mariaelenamura@uniss.it (M.E.M.); mamurgia@uniss.it (M.A.M.); 2 Dipartimento di Agraria, University of Naples Federico II, 80055 Napoli, Italy; blaiotta@unina.it

**Keywords:** sheep meat, fermented sausage, *L. plantarum*, technological characterization, Anti-*Listeria* activity, biogenic amines, decarboxylase genes, multi-copper oxidase

## Abstract

The aim of this work was to identify and characterize, from a technological and safety point of view, the lactic acid bacteria (LAB) isolated from traditional sheep-fermented sausage. First, LABs were identified then were screened for some technological parameters such as acidifying and growth ability, proteolytic and lipolytic activity and for antimicrobial activity. Finally, biogenic amine production and degradation abilities were also evaluated. This research reveals the predominance of *Lactiplantibacillus* (*L.*) *plantarum* on LAB community. Almost all *L. plantarum* strains were active against *Listeria monocytogenes* strains (inhibition zone diameters > 1 cm). None of the tested strains were positive in histidine (*hdc*A), lysine (*ldc*) and tyrosine (*tyrdc*) decarboxylase genes and only one (*L. plantarum* PT9-2) was positive to the agmatine deiminase (*agdi*) gene. Furthermore, given the positive results of the *sufl* (multi-copper oxidase) gene detection, all strains showed a potential degradation ability of biogenic amines.

## 1. Introduction

Salami are the stuffed meat-based fermented products obtained from the striated muscles generally belonging to the pig carcass, with the addition of salt and spices, minced and mixed with pork fat in variable proportions, and stuffed into natural or synthetic casings. Mediterranean countries have an ancient tradition in the production of salami, with a variety of products that are often only recognized at regional level, whose peculiarities depend on various factors as meat type, production techniques and ripening time. These information as well as the origin of the traditional products may influence the consumers responses as demonstrated by Iaccarino et al. [1] in the case of the *soppressata* salami.

Among the salami manufactured in Sardinia (Italy) the sheep sausage plays a special role because of its typical features, whose production is probably born from the high availability of sheep meat at the end of his career. Indeed, the Sardinia region owns 42% of sheep reared in Italy and the consumption of sheep meat is the highest in the country. Among other things, sheep meat lends itself to transformation into “halal products”, whose market in Italy is constantly evolving too [2].

The technology process of sheep sausage does not differ much from that of pork sausage [3]. Different cuts of sheep meat from healthy animals are commonly used as shoulder, sirloin, and legs [4]. It is essential, however, to eliminate sheep fat as well as the connective tissue both responsible for unpleasant taste; that is the reason why in some products they are replaced with pork fat. Briefly, sheep meat is minced (approx. 1 cm diameter) and eventually mixed with pork fat (cut in small pieces). Variable amounts of salt (2.8–3%) and spices (mostly pepper and garlic) are added. In the traditional manufacturing process, that are made without starter cultures, the fermentation process is carried out by natural microflora associated with the meat, the ingredients and the manufacturing environment. The mix is left to set down overnight in a fresh environment and stuffed into the pork/sheep casings. Then sausage is smoked for up to 3–4 days and then ripened for up to 20 or 30 days.

The lactic acid bacteria (LAB) constitute the main microbial group associated with fermented sausage products. LABs are responsible for lactic acid production by lactic fermentation of glycogen/glucose and the consequent pH decreasing in the substrate. The increase of acidity, especially in the first 24 h of the process, is a fundamental prerequisite for preventing the growth of pathogenic and/or spoilage microorganisms sensitive to acidity (e.g., *Listeria* spp.), ensuring the safety and preservability of the product. Besides, microbial acidification favors sausage flavor and texture development [5]. 

Although several lactobacilli are considered safe enough to be defined GRAS (Generally Recognized As Safe) and have the Qualified Presumption of Safety (QPS) status from FDA in USA and from EFSA in Europe, respectively [6], some unhealthy features as biogenic amines (BAs) production in food, cannot be underestimated. 

BAs are organic bases with intense physiological activities in the human body. On the other hand, high concentrations of BAs in food could have implications for human health, which manifest themselves in terms of hypertensive crisis, headache, nausea; tyramine and histamine in particular can also induce allergic reactions [7,8]. Furthermore, in fermented meat products, some BAs such as putrescine and cadaverine, in the presence of nitrites can form carcinogenic compounds such as nitrosamines [9]. 

The same BAs can be considered a raw material and process hygiene indicators since their formation depends on the activity of spoilage microorganisms [10]. 

Even LAB strains are able to produce biogenic amines from the precursor amino acid through decarboxylase or deiminase activities [11,12]. 

In fermented meat, where an intensive proteolytic process occurs with free amino acid release, decarboxylase activity of *Lactobacillus* spp. is implicated in histamine, tyramine, cadaverine and putrescine production [13]. Putrescine production via an agmatine deiminase method has been also reported [14]. 

Besides investigating on the BAs production, an assessment of the BAs degrading ability was equally newsworthy. LAB can degrade biogenic amine through some enzyme synthesis such as multi-copper oxidase (MCO) [15,16,17]. 

To date, and to our knowledge, no experiments have been carried out on microorganisms isolated from Sardinian sheep sausage. The aim of this work was to identify and characterize, from a technological and safety point of view, the LAB isolated from this traditional product.

## 2. Materials and Methods

In this research, a total of 40 *Lactobacillus* spp., previously isolated from traditional sheep sausage [4] were used. All the strains were refreshed in MRS (Man Rogosa and Sharpe) medium (Oxoid, Milan, Italy) at 37 °C for 48 h, under anaerobic condition (Gas-Pack; Oxoid, Milan, Italy).

Genomic DNA was extracted from bacteria using the ArchivePure DNA Yeast and Gram-positive Bacteria Kit (5 PRIME GmbH, Hamburg, Germany), according to manufacturer’s instructions. The DNA concentration, integrity and purity were assessed by spectrophotometric measurements using SPECTROstar Nano Microplate Reader (BMG Labtech, Ortenberg, Germany) and by agarose gel (1%) electrophoresis.

### 2.1. Bacteria Genotyping and Identification

Both for RAPD (Random Amplification of Polymorphic DNA) and REP-PCR (Repetitive Element Palindromic-Polymerase Chain Reaction), the amplification reaction was performed in 50 μL volume, containing 5 mM MgCl_2_, 200 μM DNTPs, 100 ng genomic DNA and 1.25 U TAQ DNA polymerase (Invitrogen). M13 primer was used in RAPD [18], whereas GTG5 primer was used for REP-PCR [19], The cycling conditions for RAPD and REP-PCR differ in annealing time and temperature. The reaction mix was incubated for 5 min at 94 °C and then amplified for 40 cycles consisting in 1 min at 94 °C, 40 s at 45 °C (RAPD) or 90 sec at 94 °C, 1 min at 40 °C (REP-PCR) and 2 min at 72 °C followed by 10 min at 72 °C. PCR products were separated by 1.5% agarose gel electrophoresis, stained by SYBR safe (Invitrogen) and visualized with Chemi Doc XRS imaging system (BioRad Laboratories, Milan, Italy). Cluster analysis of the band profiles obtained from RAPD and REP-PCR analysis was performed using the InfoQuest FP Software (version 4.5; Bio-Rad Laboratories, Hercules, CA, USA). A similarity matrix of bacterial banding profiles was calculated using Pearson’s correlation similarity coefficients. Cluster analysis of the single and combined RAPD and REP-PCR band profiles was performed using the unweighted pair-group method with arithmetic averages (UPGMA). Different bacterial strains were distinguished as those with an arbitrary cluster cut-off value of 85%. Strains associated to the same cluster were used for the identification based on 16S rRNA sequencing and *rec*A multiplex PCR assays.

A fragment of 16S rDNA gene was amplified by PCR using the following primers: W001 (5′-AGAGTTTGATCMTGGCTC-3′) and W002 (5′-GNTACCTTGTTACGACTT-3′). PCR reaction was performed as described before and conducted as follows: 4 min at 96 °C 35 cycles of 40 s at 96 °C, 30 s at 50 °C, 1 min at 72 °C and 10 min at 72 °C of final extension. Sequencing reactions were performed by Macrogen Europe (Amsterdam, The Netherlads).

Species-specific *rec*A (DNA recombination/repair protein A) gene-based primers were used for a multiplex PCR for simultaneous distinction of *L. plantarum*, *L. paraplantarum* and *L. pentosus*: PlanF (CCGTTTATGCGGAACACCTA), ParaF (GTCACAGGCATTACGAAAAC), PentF (CAGTGGCGCGGTTGATAT) and pREV (TCGGGATTACCAAACATCAC) [20]. 

PCR was performed as follows: initial denaturation at 94 °C for 3 min, 30 cycles of denaturation at 94 °C (30 s), annealing at 56 °C (10 s), and elongation at 72 °C (30 s), and final extension at 72 °C for 5 min.

### 2.2. Technological Characterization of L. plantarum Strains

#### 2.2.1. Acidifying and Growth Activity

Both acidifying and growth activity of lactobacilli were determined on SB medium [21], a suitable medium simulating the meat substrate containing meat extract 10% (Oxoid), glucose 2% (Microbiol, Cagliari, Italy) Bactopeptone 1% (Oxoid) NaCl 2.5% (Microbiol), pH 6.5 [21]. A rate (1%) of overnight culture has been inoculated in tube containing 10 mL of SB broth incubated at 30 °C for 0, 3, 6, 9, 24, 48 h. For each time-point, the pH value was determined; whereas for lactobacilli enumeration, serial 10-fold dilutions were prepared using Ringer’s solution (Oxoid) and plated on SB agar medium. After incubation of the plates in anaerobic conditions (Gas-Pack; Oxoid) at 30 °C for 48 h, colony counts were expressed as Log CFUs (Colony Forming Units) per mL of sample ± standard deviations.

#### 2.2.2. Proteolytic and Lipolytic Activity

The proteolytic activity of lactobacilli cultures was carried out on sarcoplasmic and myofibrillar proteins extracted from sheep and pork lean meat as detailed by Mauriello et al. [22]. Both protein fractions extracted were quantified by Bio-Rad Protein assay (Bio-Rad, Milan Italy) before being used in Agar Plate Assay.

A proteolytic assessment (PA) medium, containing tryptone 5 g/L; yeast extract 2.5 g/L; glucose 1 g/L, agar 15 g/L pH 6.9 and supplemented with 0.5 mg/mL protein extracts was prepared and poured into Petri dishes. After solidification, 6 mm wells were punched and filled with 50 μL of an overnight or 24 h culture for LAB and incubated for 48 h at 30 °C. A staining solution (0.1%) Comassie Blue R250, 40% methanol, 10% acetic acid was poured into Petri dishes for 1 h, and then distained to appreciate the clear zones around the wells indicating proteolytic activity [23].

Lipolytic activity was determined by agar plate method on MRS Agar (Microbiol, Cagliari, Italy) added of 1% (*v*/*v*) of tributyrin or Tween 80. The overnight culture of each *Lactobacillus* strain was first centrifuged and then resuspended in phosphate buffer at pH 7, and finally a rate of 10 µL was spotted on the surface of the plate. After 48 h of incubation at 30 °C, the opaque halo around each spot was measured. The lipolytic activity by titration was carried out as described by other authors [19]. Briefly, 1 mL of an overnight culture was inoculated in 10 mL of YTF broth medium containing 1% tryptone, 0.5% yeast extract, 3% NaCl, pH 7 and added with 4% (*w*/*v*) pork fat. After 7 days at 30 °C, to extract the lipids, the broth culture was added with 10 mL of petroleum ether and stirred for 2 min. After a short break, the upper phase was taken and titrated with 0.1 N NaOH in ethanol using phenolphthalein 1% as indicator. The lipolytic activity was expressed as grams of palmitic acid released from 100 g of total fat.

#### 2.2.3. Anti-*Listeria* Activity of *L. plantarum* Strains 

The antibacterial activity of lactobacilli was evaluated against *Listeria monocytogenes* LE STAA (E) and *Listeria monocytogenes* DSM 20600 (D) by Agar Spot Test and Agar Well Diffusion methods [24]. Briefly, a suspension of Listeria strain was inoculated (3% *v*/*v*), in the BHI agar soft (0.75% agar, Oxoid, Milan, Italy) and after medium solidification 10 µL of lactobacilli were spotted on the plate. The plates were incubated in aerobic conditions at 30 °C per 24 ore and only inhibition zone diameters > 1 cm were deemed positive. Then, the cell free supernatant (CFS) of the positive strains was adjusted to pH 6.5 and treated with catalase (1 mg mL^−1^) with the purpose of eliminate the potential inhibition due to organic acids and H_2_O_2_, respectively.

### 2.3. Proof of Strains Relevant Safety Properties

#### 2.3.1. Determination of Amino Acids Decarboxylase Activity 

LAB decarboxylase activity was determinate using a method described by Mete et al. [25]. In the medium (5 g tryptone, 8 g meat extract, 4 g yeast extract, 0.5 g tween-80, 0.2 g MgSO_4_, 0.05 g MnSO_4_, 0.04 g FeSO_4_, 0.1 g CaCO_3_ and 0.06 g bromocresol purple as pH indicator), corresponding amino acids (L-histidine, L-tyrosine, L-ornithine, L-arginine, L-lysine, L-tryptophan and L-phenylalanine) (Biochemica) were added individually at a 0.5% final concentration. By this way, an individual medium broth tube was prepared for each amino acid. The pH was adjusted to 5.3 aseptically. MRS broth cultures (30 °C, 48 h) were inoculated into decarboxylase medium without amino acids and tubes were incubated at 30 °C for 5 days. The test was carried out in 96-microtitre plates and 20 μL of each culture was inoculated into 200 μL of the same medium with corresponding amino acids in triplicates and, incubated at 30 °C for 3 days. The decarboxylase medium without amino acid was used as control (yellow color). Color conversion from yellow to purple was evaluated as amino acid decarboxylase positive. 

#### 2.3.2. Detection of the Genes Related to Biogenic Amines (BAs) and BAs Degradation Ability

PCR was used to detect the genes coding for the decarboxylase enzymes involved in the production of BAs and the gene coding for the amine oxidase degrading BAs. Primers used to detect the BAs-related genes, histidine decarboxylase (*hdc*A), tyrosine decarboxylase (*tyrdc*), agmatine deiminase (*agdi*), lysine decarboxylase (*ldc*) multi-copper oxidase (*suf*I); PCR were performed as described before, using the relevant annealing temperature (Table 1).

### 2.4. Statistical Analysis

Growth and acidification data were analyzed using the one-way ANOVA or Tukey method (*p* < 0.05) in case of significant differences compared to the control. Statistical analysis was performed using MINITAB^®^ software (Version 16.1.0, Minitab, State College, PA, USA).

## 3. Results

### 3.1. Bacteria Identification and Genotyping

**Figure 1:**
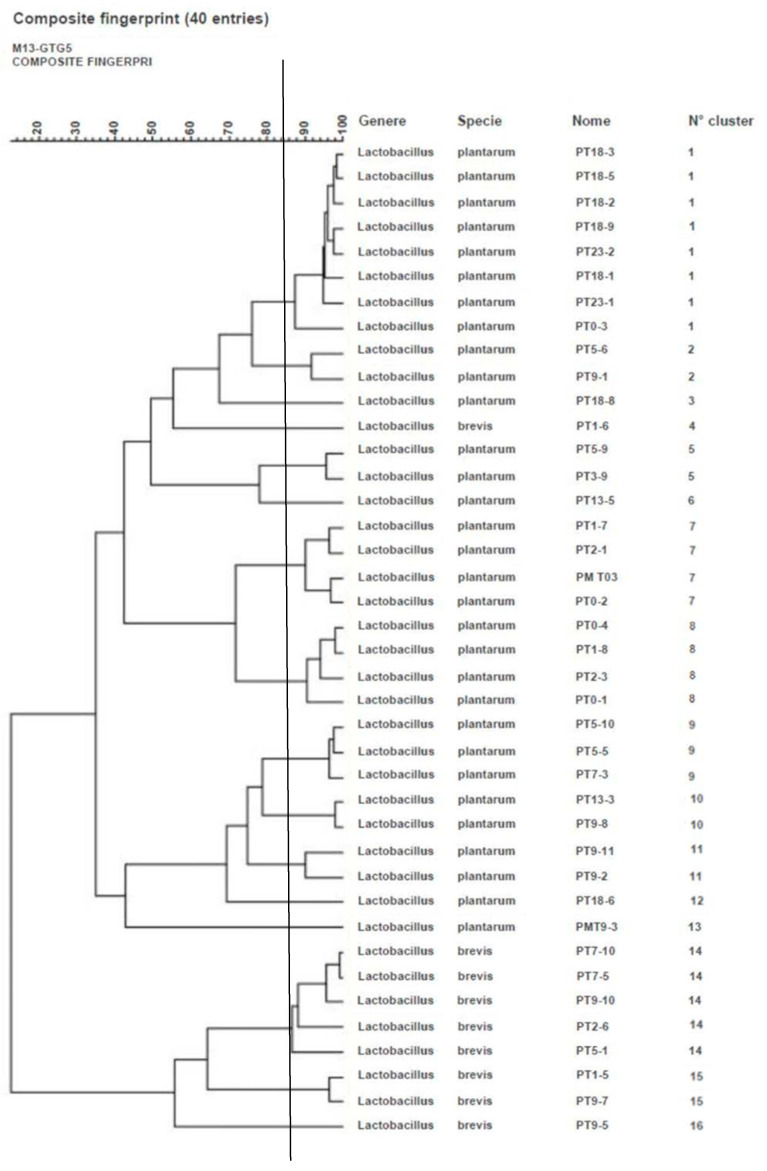
Dendrogram showing relationships among the 40 *Lactobacillus* spp. isolated from sheep sausage based on RAPD-PCR and rep-PCR banding profiles.

The analysis RAPD-PCR and rep-PCR banding profiles allowed to group 40 LAB isolates into 16 clusters, five of which were singletons (Figure 1). The 16S rRNA gene sequencing, of representative isolates of each cluster, allowed us to identify nine *L. brevis*, recently reclassified as *Levilactobacillus brevis* [30]. The remaining 31 strains, identified as Lactobacillus ssp. by 16S rDNA gene sequencing, could be identified by multiplex recA-PCR assay as *L. plantarum*, recently reclassified as *Lactiplantibacillus plantarum* [30]. Eight out of nine strains of *L. brevis* clustered closely in clusters 14, 15 and 16. A major diversity was shown in the *L. plantarum* strain; in fact, the 31 strains were spread in 12 different clusters. 

### 3.2. Technological Characterization of L. plantarum Strains

The evaluation of the technological features was carried out on 14 *L. plantarum* strains, representative of the clusters obtained based on genetic analysis. 

#### 3.2.1. Acidifying and Growth Ability of *L. plantarum* Strains

In Table 2, the data about the growth and the acidifying ability of *L. plantarum* strains are summarized. Overall, the strains grow and acidify the substrate quickly. During the first 6 h of incubation PT7-34 strain showed the highest cell counts (about 8 Log), followed by PT9-8, PT9-3 and PT9-11 strains. PT9-3 strain showed the best acidifying activity because it reduced the pH until 5.09. After 6 h, all strains went on to grow and acidify the substrate. After 24 h most strains reaching a viable count of about 9–10 log CFU mL^−1^, except for PM-T03, PT1-8 and PT23-1 strains. At the same time-point the reduction of pH is evident for PT18-8 strain (about 3.97).

#### 3.2.2. Proteolytic and Lipolytic Activity of *L. plantarum* Strains

**Figure 2:**
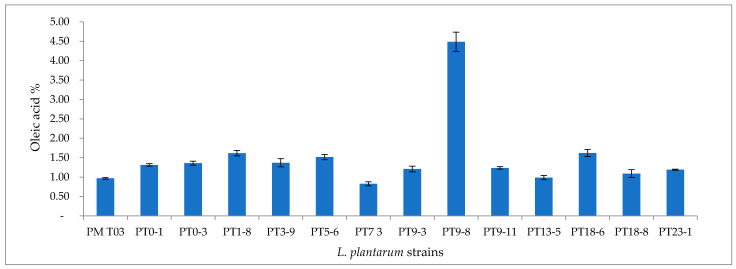
Lipolytic activity (% of oleic acid) of *L. plantarum* strains on pork fat evaluated by titration method.

*L. plantarum* strains didn’t show any proteolityc activity on both sheep and pork protein. Regarding lipolytic activity, all strains tested were unable to hydrolyze Tween 80 and tributyrin using the plate method (no inhibition halo was evaluated). Instead, some strains have been able to hydrolyze pork fat evaluated by titration method (Figure 2). In total, 11 *L. plantarum* strains produced oleic acid in quantities greater than 1%, with the best performance of the *L. plantarum* PT9-8 strain which produced 4.49% of oleic acid (Figure 2). 

### 3.3. Anti-Listeria Activity 

**Figure 3:**
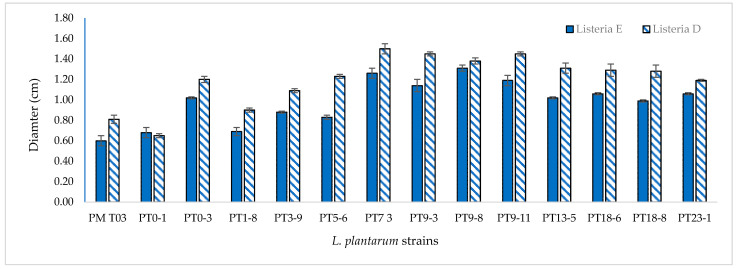
Anti-*Listeria* activity of *L. plantarum* strains by Agar Spot Test.

Anti-*Listeria* activity performed by Agar Spot Test showed almost all *L. plantarum* strains were active against both L. monocytogenes strains (ø > 1 cm) (Figure 3). However, when the possible effects of acidity and hydrogen peroxide have been eliminated, by applying the Agar Well Diffusion method, any positive results have been confirmed (ø < 1 cm).

### 3.4. Safety-Related Properties

#### 3.4.1. Amino Acid Decarboxylase Activity 

By Appling the method described by Mete et al. [25] to evaluate the decarboxylase activities of LAB isolates only two strains showed this activity: PT9-2, was found to possess decarboxylate activity on lysine and arginine; PT-03 on lysine only (Table 3). 

#### 3.4.2. Detection of BA-Producing Genes of the Strains

**Figure 4:**
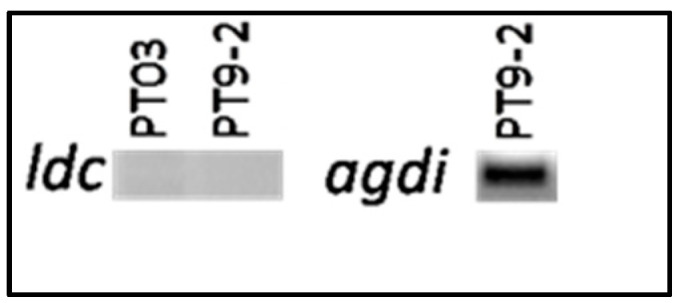
PCR related to biogenic amines (BAs) production. PCR products of the lysine decarboxylase (ldc) and agmatine deiminase (agdi) genes.

In order to confirm the result obtained in the amino acid decarboxylase activity assay, PCR methods were used to verify the presence of four genes involved in the production of the main BAs: three genes codifying for decarboxylase enzymes (hdc, tyrdc, and ldc) and one for agmatine deiminase (agdi). None of the tested strains were positive in histidine decarboxylase gene (hdcA), lysine decarboxylase gene (ldc) (Figure 4) and tyrosine decarboxylase gene (tyrdc), indicating that the tested strains might not have cadaverine and histamine formation ability. However, in strain PT9-2, the presence of the agmatine deiminase gene was confirmed by PCR (Figure 4).

#### 3.4.3. BAs degradation ability

**Figure 5:**
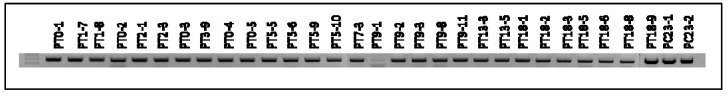
PCR detection of multi-copper oxidase gene (sufI).

In this study, SufI primer pair was used to detect the multi-copper oxidase gene, since in LAB the multi-copper oxidase showed a high ability of degrading BAs [16]. All strains exhibited positive results with primers designed for sufI gene and the lengths of the corresponding PCR products were about 454 pb (Figure 5). This result demonstrates that all strains probably possessed the multi-copper oxidase for degrading BAs. Notable differences in signals are due to a different starting material amount. 

## 4. Discussion

Sheep sausage is one of the traditional products identified the Sardinian agro-pastoral sector. As the enhancement of a fermented food cannot be separated from the knowledge of the microorganisms involved in the process, this study focuses on the identification and characterization of the dominant LAB species isolated from sheep sausage.

This research reveals the predominance of *L. plantarum* on LAB community in Sardinian sheep sausage. *L. plantarum* is considered a plant/vegetal-associate bacteria, that probably reflects the high presence of *L. plantarum* in many Sardinian dairy and meat fermented product [3,4,31], traditionally manufactured by raw material (milk or meat) derived from sheep bred in pasture. This could explain the clear predominance of *L. plantarum* in the traditional sheep sausage produced in Sardinia.

Regardless, *L. plantarum* is a versatile species with an adaptive ability for many different conditions [32] as salt and low pH allow it to colonize both animal and plant origin products. *L. plantarum* is commonly present in fermented meat products in many of which, among the lactobacilli, it is the most prevalent species [33,34,35,36] albeit other species as Lb. *sakei* and Lb. *curvatus* in particular have been identified [37,38,39]. Alongside *L. plantarum* only *L. brevis* has been isolated. The occurrence of obligate heterofermentative LABs as *L. brevis* in fermented products has been reported in generic fermented meat [40] even if their use in the process is not as desirable as some metabolites produced, such as CO_2_ gas and acetic acid, and can lead to the formation of air holes inside the sausage conferring an unpleasant taste [41]. For these reasons only bacteria belonging to the *L. plantarum* species were tested for technological and safety features.

Growth and acidifying activities are the main ability required of LAB to be used as a starter culture, as a high number of viable microorganisms as well as a drop of pH in the first part of the fermentation stage allow to counteract development of spoilage and/or pathogen bacteria through competition for nutrients or antimicrobial metabolite production (e.g., organic acid, bacteriocins).

*Listeria monocytogenes* is a pathogenic microorganisms can easily contaminate and/or re-contaminate fermented meat products [42] and it is capable of develop during ripening process, especially in the meat products characterized by values of pH > 4.5–5 as Sardinian sheep sausage [4,43]. Employment of *L. plantarum* as a natural preservative against Listeria in sausage production has been reported in Greek [44] and Turkish [45] fermented sausage.

In this study, the Anti-*Listeria* activity of *L. plantarum* strains was probably due to acidification [46,47] and depending on both *Lactobacillus* and pathogenic strains [48]. Interestingly, *L. plantarum* PT23-1 resulted active against a honey bee pathogen *Paenibacillus* larvae and a possible role of small antimicrobial peptides has been supposed [49].

In fermented sausage, proteolysis and lipolysis are the main biochemical processes due to the action of both endogenous meat and microbial enzymes and, characterize many traits of ripened product as texture and flavor. Despite micrococci and staphylococci being the main bacteria that contribute to the ripening process, a certain enzymatic activity by *L. plantarum* strains on sarcoplasmic proteins from different sources has been determined [50,51], which is in contrast to our results as all *L. plantarum* strains did not show any proteolityc activity on both sheep and pork protein. Controversial results of *L. plantarum* lipolytic activity have also been reported: a negative lipolytic activity on pork fat evaluated by different methods [23,34], while some strains isolated from camel sausage revelated the ability to hydrolyze camel and beef fat [52].

High concentrations of BAs are an unhealthy trait of fermented sausage, although the European law [53] reports only histamine as a safety criterion in some fish products. The main BAs reported in generic fermented sausage are tyramine, tryptamine, cadaverine, putrescine and histamine [13]. Accumulation of BAs in fermented meat is difficult to avoid because fermentation conditions cannot be easily modified [12]. In addition, the technological process does not provide any heat treatment useful to reduce the spoilage microorganisms, potential producers of BAs.

These results points out a weak abilities of the isolated *L. plantarum* to produce BAs in agreement with others authors [54,55,56] as well as a potential degrading BAs ability.

The control of microorganisms involved in BAs content can be a way to guarantee fermented sausage safety, both by applying good hygiene practices and by using starter cultures that do not produce BAs and/or able to reduce their level.

## 5. Conclusions

The study of the microbial community of fermented foods is an essential condition for knowing the microbial species characterizing the product and for selecting the species/strains to be used as a starter culture. In this research, the predominance of *L. plantarum* on the LAB community of fermented sheep sausage on the one hand, indicates low biodiversity, on the other hand, shows the ability of *L. plantarum* to colonize the outdoor natural environment and as in this case the entire sheep meat processing chain.

Overall, *L. plantarum* strains analysis showed adequate technological and safety characteristics to be used as starter, alone or mixed with other non-LAB microorganisms, a strategy useful to improve the quality of the fermented sausage and maintain its typicality.

To the best our knowledge, this is the first microbiological study on Sardinian fermented sausage made with sheep meat, an initial step for future study of the entire microbial population.

## Figures and Tables

**Table 1:** Lists of primers to detect the genes involved in the formation and degradation of BAs.

GENE	PRIMER FORWARD	PRIMER REVERSE	T°	bp	Reference
***hdc***	5′AGATGGTATTGTTTCTTATG3′	5′AGACCATACACCATAACCTT3′	46	367	[26]
***tyrdc***	5′CAAATGGAAGAAGAAGTTGG3′	5′ACATAGTCAACCATATTGAA3′	50	1100	[27]
***ldc***	5′TAGGTTCAGATTGGCCCTTAG3′	5′ACTTCAACACCTGCTGCTTTC3′	52	769	[28]
***agdi***	5′ATGCCCGGTGAATTTGAA3′	5′ TTGCGCTGGTTTAGCAC3′	48	90	[29]
***SufI***	5′TCGTTGATTTTGGTCAGTATCA3′	5′ATATGGCAGTGATACATGTAAAC3′	50	454	* NC_004567.2

*hdc,* Histidine decarboxylase; *tyrdc*, tyrosine decarboxylase; *ldc,* lysine decarboxylase; *agdi,* agmatine deiminase; *SufI,* multi-copper oxidase. Note: T° is temperature of annealing used for PCR; bp is the expected amplicon size; *, Accession number.

**Table 2:** Acidifying ability (pH) and growth ability (log CFU mL^−^^1^) of 14 *L. plantarum* strains at different time (0, 3, 6, 9, 24 h) in SB medium.

*L. plantarum*	0 h		3 h		6 h		9 h		24 h	
	pH	Log CFU mL^−1^	pH	Log CFU mL^−1^	pH	Log CFU mL^−1^	pH	Log CFU mL^−1^	pH	Log CFU mL^−1^
PM T03	5.88 ± 0.01 ^a^	4.75 ± 0.01 ^a^	5.62 ± 0.02 ^a^	5.53 ± 0.01 ^a^	5.54 ± 0.01 ^b^	5.63 ± 0.02 ^b^	4.87 ± 0.02 ^a^	6.27 ± 0.01 ^b^	4.27 ± 0.03 ^a^	7.99 ± 0.02 ^b^
PT0-1	5.87 ± 0.01 ^a^	4.75 ± 0.01 ^a^	5.63 ± 0.04 ^b^	5.38 ± 0.03 ^a^	5.57 ± 0.01 ^b^	5.34 ± 0.02 ^a^	5.47 ± 0.03 ^b^	6.21 ± 0.00 ^b^	4.28 ± 0.01 ^b^	7.85 ± 0.05 ^a^
PT0-3	5.87 ± 0.03 ^a^	4.52 ± 0.06 ^a^	5.22 ± 0.02 ^a^	5.36 ± 0.03 ^a^	5.29 ± 0.01 ^b^	6.18 ± 0.08 ^b^	4.85 ± 0.06 ^a^	6.59 ± 0.08 ^b^	4.14 ± 0.02 ^b^	6.53 ± 0.05 ^b^
PT1-8	5.84 ± 0.05 ^a^	4.80 ± 0.02 ^a^	5.79 ± 0.03 ^b^	5.54 ± 0.01 ^a^	5.16 ± 0.04 ^b^	5.46 ± 0.02 ^b^	5.04 ± 0.06 ^b^	6.33 ± 0.03 ^a^	3.94 ± 0.04 ^b^	7.75 ± 0.06 ^a^
PT3-9	5.86 ± 0.01 ^a^	4.52 ± 0.01 ^a^	5.63 ± 0.02 ^b^	5.17 ± 0.04 ^a^	5.44 ± 0.04 ^a^	4.99 ± 0.03 ^b^	4.67 ± 0.06 ^b^	6.09 ± 0.04 ^b^	4.05 ± 0.04 ^b^	6.64 ± 0.08 ^b^
PT5-6	5.80 ± 0.03 ^a^	4.55 ± 0.02 ^a^	5.41 ± 0.05 ^b^	5.20 ± 0.01 ^a^	5.20 ± 0.04 ^a^	5.20 ± 0.02 ^a^	5.18 ± 0.03 ^a^	6.45 ± 0.05 ^a^	4.25 ± 0.03 ^a^	7.67 ± 0.02 ^b^
PT7-3	5.74 ± 0.00 ^a^	4.79 ± 0.00 ^a^	5.71 ± 0.04 ^b^	5.44 ± 0.01 ^a^	5.40 ± 0.01 ^b^	5.36 ± 0.01 ^b^	5.09 ± 0.03 ^a^	8.31 ± 0.02 ^b^	4.27 ± 0.04 ^a^	9.44 ± 0.03 ^b^
PT9-8	5.81 ± 0.03 ^a^	4.95 ± 0.02 ^a^	5.71 ± 0.01 ^a^	5.53 ± 0.01 ^a^	5.49 ± 0.02 ^a^	5.46 ± 0.01 ^b^	5.12 ± 0.06 ^b^	7.33 ± 0.01 ^b^	4.32 ± 0.03 ^a^	8.72 ± 0.01 ^b^
PT9-11	5.83 ± 0.03 ^a^	4.82 ± 0.01 ^a^	5.80 ± 0.00 ^b^	5.38 ± 0.00 ^a^	5.30 ± 0.02 ^b^	5.32 ± 0.01 ^b^	5.14 ± 0.01 ^b^	7.67 ± 0.03 ^b^	4.25 ± 0.01 ^a^	8.84 ± 0.01 ^b^
PT9-3	5.86 ± 0.02 ^a^	4.93 ± 0.03 ^a^	5.63 ± 0.01 ^a^	5.64 ± 0.00 ^a^	5.09 ± 0.01 ^a^	5.30 ± 0.01 ^b^	5.08 ± 0.04 ^a^	7.29 ± 0.00 ^b^	4.31 ± 0.04 ^a^	9.58 ± 0.05 ^b^
PT13-5	5.81 ± 0.02 ^a^	4.43 ± 0.01 ^a^	5.47 ± 0.02 ^b^	5.30 ± 0.03 ^a^	5.41 ± 0.03 ^b^	5.26 ± 0.04 ^a^	4.91 ± 0.01 ^b^	6.61 ± 0.07 ^a^	4.12 ± 0.02 ^a^	7.29 ± 0.04 ^a^
PT18-6	5.86 ± 0.03 ^a^	4.87 ± 0.03 ^a^	5.47 ± 0.03 ^b^	5.83 ± 0.07 ^a^	5.31 ± 0.05 ^a^	5.62 ± 0.03 ^b^	4.47 ± 0.03 ^b^	6.90 ± 0.04 ^b^	4.09 ± 0.02 ^a^	7.86 ± 0.05 ^a^
PT18-8	5.85 ± 0.05 ^a^	4.50 ± 0.03 ^a^	5.51 ± 0.01 ^b^	5.32 ± 0.01 ^a^	5.42 ± 0.08 ^a^	5.10 ± 0.02 ^b^	4.87 ± 0.04 ^b^	6.04 ± 0.10 ^a^	4.05 ± 0.06 ^a^	7.07 ± 0.03 ^b^
PT23-1	5.81 ± 0.06 ^a^	4.45 ± 0.05 ^a^	5.49 ± 0.05 ^a^	5.27 ± 0.02 ^a^	5.43 ± 0.07 ^b^	5.12 ± 0.02 ^b^	4.69 ± 0.08 ^b^	5.95 ± 0.03 ^b^	3.82 ± 0.05 ^b^	6.67 ± 0.04 ^b^

Data represent the mean (±standard deviation, SD) of three independent experiments. Different letters indicate significant differences (*p* < 0.05) among different time for the same strain.

**Table 3:** Amino acid decarboxylase activity test results of *L. plantarum* strains.

*L. plantarum* strains	Hys	Lys	Arg	Orn	Tyr	Trp	Phe
PT0-3	−	+	−	−	−	−	−
PT9-2	−	+	+	−	−	−	−

His: histidine; Lys: lysine, Arg: arginine; Orn: ornithine; Tyr: tyramine; Trp: Tryptamine; Phe: phenylalanine.

## Data Availability

All relevant data of this study are presented. Additional data can be provided upon request.
